# Self-management experiences in fall prevention among community-dwelling older adults in China: a descriptive qualitative study

**DOI:** 10.1080/07853890.2024.2392878

**Published:** 2024-09-25

**Authors:** Yu-ting Yang, Miao Yao, Yong-wei Yang, Ting Lin

**Affiliations:** The School of Nursing, Fujian Medical University, Fuzhou, China

**Keywords:** Community-dwelling, qualitative research, falls, older adults, prevention, self-management

## Abstract

**Background:**

Falls are the most common injuries in older adults, and fall prevention is one of the primary measures to achieve healthy aging. Self-management refers to the measures taken by individuals to avoid various adverse factors and health damage to protect and promote their health. This study aimed to explore the factors and measures of self-managed fall prevention among community-dwelling older adults.

**Methods:**

A qualitative study based in two communities under the jurisdiction of Ninghua Street and Shanghai Street was conducted in Fuzhou, China. Semi-structured and face-to-face individual interviews were conducted with 15 community-dwelling older adults. Interviews were conducted by the first and second authors who had participated in qualitative training and were audio-recorded and transcribed. The data were analysed deductively with content analysis.

**Results:**

The research revealed two themes with associated sub-themes: 1) influencing factors of self-managed fall prevention, and 2) promoting self-managed measures to prevent falls.

**Conclusions:**

Individual, social support, community advocacy, and road condition influenced self-managed fall prevention. Active exercise, adjusting home environment and clothing, and multi-channel acquisition of self-managed fall prevention knowledge can reduce the incidence of falls among older adults. Identifying these experiences will help older adults improve their awareness of preventing falls, take responsibility for themselves, and reduce the incidence of falls.

**Trial registration:**

Chinese Clinical Trial Register: ChiCTR2200060705; reg. date: June 8, 2022.

## Introduction

China has one of the fastest-aging populations [1]. According to the Seventh National Census [2], 264 million adults aged 60 and older account for 18.7% of the country’s population. Falls are the most common and primary unintentional injuries in older adults [3]. With increasing age, the incidence of falls gradually increases. According to data from the World Health Organization [4], 28–35% of adults over age 65 have fallen at least once a year, and the incidence of falls among adults over the age of 70 is 32–42%. The incidence of falls among people aged over 80 is as high as 50% [5]. A meta-analysis in 2022 [6] reported that the incidence of falls among older adults in China was 19.3%. Falling causes physical injuries such as soft tissue bruises and fractures and psychological problems such as anxiety, reduced balance confidence, and fear of falling, resulting in the loss of functional independence, reduced exercise, avoidance of social activities [7,8], and even death [9]. Worldwide, approximately 684,000 people die from falls each year, with the latest proportion of deaths occurring among people aged 60 and older [9]. Falls are the leading cause of death from injury among older adults aged 65 and above in China, and they are also the primary cause of traumatic fractures in older adults [10]. More than half of older adults who seek treatment in medical institutions for injury are there because of falls [10]. Additionally, falls can cause a heavy economic burden on families and the society [8,11–13]. Falls are harmful to older adults and can seriously affect their quality of life.

Although older adults have a high incidence of falls, falls are not inevitable owing to aging but can be prevented and controlled [14,15]. In the 2015 World Report on Aging and Health, the World Health Organization [16] defined healthy aging as the process of developing and maintaining the functions necessary for the healthy life of older adults and fall prevention among community-dwelling older adults is a primary requirement to achieve healthy aging [17]. In a systematic fall prevention project for older adults, the participation and efforts of individuals, families, medical staff, and society as a whole is required, and older adults play an important role [14]. However, Huang [18] found that fall prevention practice, whether in hospitals, communities, or nursing homes, focuses on the community, nursing homes, hospitals, and medical staff to improve facilities and equipment, system management, work service processes, or intervention measures to promote fall prevention. These studies often ignore the primary role of older adults in fall prevention and subjective initiative, focusing on the role of passive cooperation [18–22]; therefore, the enthusiasm and compliance of older adults to participate fall prevention is low, and effectiveness is limited.

Through self-management, individuals can promote disease treatment, rehabilitation, and maintain health [23]. Self-management research can be traced back to psychobehavioural therapy in the 1960s [24]. Self-management emphasises ‘people-oriented’ measures to avoid adverse factors and health damage to protect and promote their health [25]. Self-management has been widely used in different fields, especially in health education programs for patients with chronic diseases, such as hypertension [26], diabetes [27], and stroke [28]. Self-management is more effective than passive participation in disease management. Currently, falls are regarded as preventable chronic diseases worldwide [29], and prevention is emphasised in controlling the incidence of falls [30]. Schnock et al. [31] defined the self-management of fall prevention as individuals taking actions or behaviours to prevent falling. Huang [18] defined self-managed fall prevention for older adults as active participation with the assistance of medical personnel in the assessment of fall risk factors, identification of fall hazards, and responses to fall hazards to maintain their safety and avoid fall injuries. However, existing prevention measures include behaviour restriction or instilling knowledge of fall prevention in older adults, instead of providing them with training in fall prevention to change risky behaviours and ignoring human subjective initiative altogether [18]. Additionally, few studies have explored the influencing factors and improvement measures for self-managed fall prevention from the perspective of older adults.

Fall prevention is a primary measure to achieve healthy aging; however, measures to prevent falls are primarily implemented by healthcare workers. Thus, older adults are in a passive cooperative position. The concept of self-management emphasises that individuals are at the centre and maintain their health through their own efforts and has demonstrated favourable results in studies on managing various chronic diseases. Few studies have focused on fall prevention among community-dwelling older adults. Self-managed fall prevention considers older adults as the main body, emphasises their responsibility in maintaining their health and confidence in active responses, and pays attention to skills training and behaviour, which is a new concept geared towards older adults so they can prevent falls. Therefore, this study aimed to explore the factors and measures for self-managed fall prevention among community-dwelling older adults. Our study reveals two important themes related to fall prevention: influencing factors and promotion of self-managed measures.

## Methods

### Study design

This is a descriptive qualitative study [32]. In an ideal situation, researchers and participants have direct communication and interaction. In the process of conversation, the researcher deeply explored the participants’ thinking perspectives and their subjective world from multiple angles. Then, the collected data and information are fully summarized and sorted out. We used qualitative methods to better understand the factors and measures of self-managed fall prevention from the perspective of older adults [33]. We used EQUATOR’s COREQ checklist [34] to check for this qualitative study (Supplementary File 1).

The Health Belief Model (HBM) is a theoretical framework of this study. The HBM explained individuals’ engagement in preventive health behaviour as follows. (1) perceived susceptibility of the health condition, (2) perceived severity of the health condition, (3) perceived benefits to taking the recommended action, (4) perceived barriers to taking the recommended action, (5) cues to action to engage in the preventive-health behaviour, and (6) self-efficacy to engage in the prevention-health behaviour [35]. The HBM is one of the most widely used theoretical frameworks to plan and design fall prevention strategies [36].

### Participants and recruitment

The study population included community-dwelling older adults in China. Inclusion criteria included age 60 and above, living in urban communities for six months or more, and the ability to express and comprehend simple Chinese characters. Exclusion criteria included having a hearing impairment, speech impairment, severe cognitive impairment, mental illness, and other severe or terminal diseases. Researchers judged whether participants met the inclusion criteria and whether to exclude them. Researchers contacted older adults who conformed to the inclusion criteria, asked if they would like to participate in this study, and provided them with written information for their consideration. The study involving human participants was reviewed and approved by the Ethics Review Committee of Fujian Medical University (No.: 2022/00074). In the meantime, our study adhered to the Declaration of Helsinki.

From September 1 to October 28, 2022, convenient sampling was conducted to select two communities under the jurisdiction of Ninghua Street and Shanghai Street, Taijiang District, Fuzhou City. When older adults in the two communities came to the Ninghua Street and Shanghai Street Community Health Service Centers for medical examinations, they would see the recruitment poster. When they asked about it, the team members would approach the participants, introduce this study, and leave them with contact information and three days of time for them to consider whether they would like to participate. Then, through purposive sampling, 15 participants who met inclusion criteria and could provide rich information were selected as research objects, numbered N1–N15. The first and second authors, having master’s degrees in nursing and received qualitative research training, conducted interviews with participants. They introduced themselves to older adults as geriatric nursing researchers. Researchers conducted face-to-face semi-structured interviews with older adults. All participants were made aware of the purpose, significance, methodology, and voluntary nature before interviews. Each participant signed an informed consent form. The interviewers assured participants the research team would guard confidentiality and that only the researchers on the team could access it. We confirmed that written consent to publish these individual quotes has been obtained from all individuals (or their legal guardian). We took every effort to anonymize individuals.

### Data collection

The interview process was recorded with the consent of the participants. The interviews were transcribed verbatim in Chinese. We interviewed them in a quiet location, including their home or nearby park setting. Researchers conducted 30–40 min semi-structured interviews to obtain information about self-managed fall prevention with various interview techniques including questioning, response, repetition, reorganization, and summary. In addition, researchers adjusted the interview strategy in a timely manner according to the situation. Handwritten records on the spot supplemented verbal or non-verbal information to ensure interview data were actual, complete, accurate, and comprehensive to reflect the views that older adults wanted to express. In the meantime, we collected their demographics. The HBM’s six constructs informed the approach to the semi-structured interview guide, which was uploaded as a supplementary file (Supplementary File 2). Older adults received a gift as compensation for study participation. The first author translated the quotes for the manuscript. To ensure the accuracy of the translation, we submitted the quotes and the translation manuscript to an editor with a Chinese and English mother tongue. After it was returned, the first author confirmed the translation again.

### Data processing and analysis

We collected data and analysed data simultaneously. Within 24 h after each interview, the researchers carefully listened to the recordings and transcribed the interview recordings word for word and sentence for sentence into written materials. The researcher recorded the pauses, tones, emotional expressions, and body language in the conversation and wrote memos. After completing the transcriptions, researchers checked the text and sent it to the participants for verification. According to Saunders et al. [37], data saturation differs depending on the type of study and assumptions concerning whether the data represent an ongoing process. After each interview, we evaluated the data to determine whether there were new patterns or variations to confirm saturation. The interviews were continued until data saturation.

We used QSR Nvivo11.0 (www.qsrinternational.com), a qualitative data analysis software, to store and encode the transcribed text and analyse the data *via* qualitative content analysis [38]. We employed an iterative-inductive, team-based coding approach to code and analyse the text [39]. Firstly, two researchers read the data repeatedly and immersed themselves to get a holistic understanding. Then, we found, extracted, and coded expressions related to the research topic sentence by sentence, and developed a codebook. We labelled the main ideas and concepts in the data and grouped similar codes into corresponding categories to form themes and sub-themes. We generated themes that involved open-ended coding of several transcripts with no predetermined codes or categories. In addition, researchers immersed themselves in the original data, read and re-read the data, and constantly compared and classified, discussed with each other, and checked and modified the codebook and the existing subject framework. The team had two meetings to update codebooks and present memos. All codes from the codebook were applied to the fifteen transcripts, then refined, reduced, and expanded. Codes were categorized into two themes and described the latent meaning of overt statements [40]. Table 1 is an example of analysing data encoding. Finally, according to the actual situation reflected in the data, we modified and improved the theme framework, forming the existing two themes, including influencing factors of self-managed fall prevention and promoting self-management measures to prevent falls. Participants’ quotes illustrate the key findings.

**Table 1.** Example of analyzing with subthemes and themes.

Meaning unit	Condensed meaning unit	Code	Subtheme	Theme
Where the indoor floor is slippery, such as when the bathroom is humid, we will choose to put non-slip mats in the bathroom. In addition, if the indoor slippers’ sole is hard and smooth, it is easy to cause us to fall. Therefore, we will avoid wearing the above slippers. We will choose soft, more non-slip shoes indoors.	Putting non-slip mats in the bathroom and wearing suitable shoes indoors	Changing the bathroom environment and home dress	Changing home environment and home dress	Promoting self-management measures to prevent falls

Trustworthiness of this study was ensured using the criteria described by Cascio et al. [39] and Lincoln and Guba [41]. Credibility was ensured by member-checking during the interview and analysis triangulation, where the two authors independently analysed the transcript and then reached a consensus about the findings. We would also ensure that appropriate and authentic citations enhance trustworthiness. Transferability was established by thoroughly describing the findings and relating them to similar studies. Dependability and confirmability were ensured by discussing the findings, reaching consensus, and maintaining a thorough audit trail.

## Results

The demographic characteristics of the participants are presented in Table 2. Researchers extracted two themes and seven subthemes of self-managed fall prevention among participants: (a) influencing factors of self-managed fall prevention (individual, social support, community advocacy, and road condition), and (b) promoting self-management measures to prevent falls (active exercise, changing home environment and home wear, and multichannel acquisition of self-managed fall prevention knowledge).

**Table 2.** Sociodemographic characteristics (*n* = 15).

Variable	Categories	*n* (*%*)
Age, year (Range 63 ∼ 88)	60 ∼ 69	2(13.33)
	70 ∼ 79	6(40.00)
	≥80	7(46.67)
Gender	Male	11(73.33)
	Female	4(26.67)
Education	Junior high school and below	2(13.33)
	High school or technical secondary school	7(46.67)
	Bachelor or college degree	6(40.00)
Marital status	Married	13(86.67)
	Widowed	2(13.33)
Living status	Living with spouse and child(ren)	6(40.00)
	Living with spouse	7(46.67)
	Living with child(ren)	1(6.67)
	Living alone	1(6.67)
Whether to fall	Yes	9(60.00)
	No	6(40.00)

### Influencing factors of self-managed fall prevention

#### Sub-theme 1: Individual

To be responsible for their health and reduce the burden on their family, older adults would pay attention to preventing falls. Most participants thought that the key to preventing falls depended on themselves and that they were primarily responsible for their health.

Older adults can prevent falls themselves. We must be careful to walk slowly on the road, not too fast. -ID8

Preventing falls is up to ourselves. Pay attention every day. If not, you will fall easily. -ID6

The individual plays the most important role because falling is an individual behaviour, and the individual needs to pay attention to it in all aspects so that such accidents do not easily occur. -ID2

Participants regarded fall prevention as a sense of responsibility towards their families and that fall prevention could reduce the burden on their families and unnecessary trouble.

As old as I am, I must learn to protect myself and not add trouble to my family with small children. For example, if I get hospitalised, it will cost money. If the pension is not enough, it will affect my children’s finances because they will have to pay for me. -ID6

Take care not to fall, or you will increase the burden on your children. It is not easy for them to make money outside. We take good care of ourselves to ease our kids’ burden. -ID3

#### Sub-theme 2: Social support

The care and reminders of family members could encourage older adults in this study to pay attention to preventing falls and was the driving force for participants to prevent falls and engage in self-management.

The family said to pay attention, walk slowly, and do not rush. My son reminds me not to fall when I go out. My children remind me. -N6

My child told me to walk carefully, watch the road, and do everything carefully to not fall. -N3

My children and wife would tell me they washed the floor with water and that I should pay attention while walking. -N4

#### Sub-theme 3: Community advocacy

Community support played a primary role in improving the self-management ability of participants to prevent falls; however, most participants said that they received less community support, including a lack of health education, life care, and spiritual care.

There are no fall prevention programs in the community to educate older adults. -N8

Our neighbourhood committee does not care about anything, let alone coming to our door to tell us how to prevent falls. -N1

There is still less community support for older people to prevent falls, and we do not talk to the community much. -N15

The community has not issued us the manual for preventing falls and so on. It does not have this function. The community also faces difficulties, and the workforce is lacking. There is a lot of work, and funds are required to do anything. The community cannot carry out the project without funds. -N3

The participants expressed hope that the community would provide healthcare education programs for fall prevention in older adults.

I do not know if my risk of falling is high or low, and I would like my community doctors and nurses to help me evaluate it and teach me how to prevent falls, but the community has not done so yet. -N6

#### Sub-theme 4: Road condition

The participants identified poor road conditions as barriers to self-managed fall prevention. Poor road conditions coupled with bad weather greatly increase the risk of falls.

It is easy for older adults to slip on maintenance hole covers, potholes, wood or stone roads, and plastic waste left on the pavement. -N6

The road construction caused the ground to become uneven. There were puddles on the ground. Older adults fell over when they walked over. Additionally, indiscriminate parking of vehicles can also cause older adults to fall. -N8

It is raining today; the road is slippery. So older adults are more likely to fall. The road is congested with traffic. We may fall if we do not realise and may be hit by a car. -N2

Now people need to drive electric bikes to deliver food. If we do not react in time, we may knock down. -N5

### Promoting self-managed measures to prevent falls

#### Sub-theme 1: Active exercise

With increasing age, the function of all aspects of the body declines, and the incidence of falls is high. Participants believed that they were more active in exercising and improving their physical fitness to prevent falls.

In general, if older adults want to avoid falling, they must do regular exercise, and the two most effective kinds of exercises are: walking and resistance exercises. -N8

Participants believed that exercise could improve physical fitness and reduce the consequences of falls.

Prevent falls, walk, and exercise more. Do not be too fat, and the body will be flexible. We should build up our fitness and keep exercising. -N14

Simultaneously, participants believed that suitable exercise and moderate-intensity exercise were better at preventing falls.

Older adults cannot do strenuous exercise. But I take a walk to improve my flexibility. -N2

I like to go outdoor for cardio. I do not do strenuous exercise. I know my body when it comes to preventing falls. -N1

#### Sub-theme 2: Changing home environment and home dress

Home was the primary place of life for older adults in this study. They gradually began to pay attention to ensure that their home environment and clothing was reasonable in terms of avoiding falls.

Older adults should be careful when bathing. They should wear non-slip slippers when taking a bath. The home environment is important. Do not put debris on the floor and clean it up to avoid tripping. Older adults should walk carefully at night and pay attention to not slip. It is necessary to hold hands. -ID14

The bathroom should be kept clean and dry. Like for the bathtub in my home, there must be non-slip mats and handrails for older adults. -ID15

There were also participants considering retrofitting their homes for aging.

Now, we are also considering whether to retrofit the floor, kitchen, and bathroom at home. -ID5

#### Sub-theme 3: Multichannel acquisition of self-managed fall prevention knowledge

Participants needed rich self-managed fall prevention knowledge to improve their self-management abilities. Students can acquire knowledge from many sources.

Self-managed fall prevention knowledge comes from mobile phone news reports or short videos. -N2

Check fall prevention articles forwarded by others via WeChat. -N4

Simultaneously, participants were also good at summarising their own life experiences.

I will sum up my experience of preventing falls. -N4

We have lived for decades and know how to prevent falls. It is basic life knowledge. -N2

The older adults in this study observed other individuals’ fall experiences to gain experience.

We will sum up the experience of people in our society. We see others looking at their mobile phone and falling. To summarise, walk without looking at the mobile phone. -N6

## Discussion

Fall-reduction behaviours in older adults is based on their sense of self-management [42]. This study’s results showed that although most older adults are aware of the severity of falls and believe that they themselves are the key to preventing them, some still have a fatalistic view of falls and believe they are unpredictable and predestined, similar to the findings of Kiyoshi-Teo et al. [43]. Community nursing staff could implement targeted intervention measures to help older adults realise the value of self-management in preventing falls and improving their self-efficacy. For older adults with weak self-management awareness of fall prevention, community nursing staff should strengthen the popularisation of knowledge, correct the wrong ideas of older adults, make them fully realise that falls can be prevented and controlled, emphasise the necessity and importance of self-management to prevent falls, and be proactive about fall prevention. For older adults to have a sense of self-managed fall prevention, community nursing staff should adopt positive encouragement strategies to affirm their consciousness and provide positive encouragement. Fall self-efficacy refers to older adults’ confidence in their ability to perform activities without falling in daily life [44]. The interviews revealed that older adults recognise the primary role of their inner strength in preventing falls and pay attention to preventing falls in daily life, indicating that they have fall efficacy. Soh et al. [45] believed that the traditional interpretation of fall efficacy needs to be reconsidered and that fall efficacy should be defined as the perceived ability to manage potential fall threats. Therefore, it is necessary to improve fall efficacy in older adults, enhance their ability to manage fall threats, and encourage them to improve their sense of fall prevention.

As emphasized by the HBM [35], acquiring knowledge is essential for understanding the susceptibility and severity of health conditions. Adequate knowledge serves as the foundation and prerequisite for implementing correct behaviours. Leahy [46] emphasized that receiving comprehensive and professional health education can assist individuals in better grasping disease-related knowledge, thereby facilitating patient disease management. Consequently, older adults must acquire knowledge and skills related to fall prevention to enhance their self-management abilities. This study reveals that older adults lack channels for obtaining self-managed fall prevention knowledge and professional resources. The knowledge they acquire is often one-sided and fragmented, potentially leading to a deficiency in professional knowledge and hindering behavioural changes. These findings align with the study conducted by Tian et al. [22]. To address this, we aim to expand the information support channels for older adults, providing professional and diverse guidance and assistance. For instance, we plan to provide recommendations to authoritative websites and public platforms dedicated to fall prevention self-management. Community workers can also distribute manuals and books to older adults, offering professional and accurate information on self-managed knowledge and skills for preventing falls.

Social support encompasses the material and spiritual assistance received by an individual from their family or other social networks [47]. This assistance originates from a range of sources, including family, relatives, friends, the community, and society at large. The social support system is a crucial determinant of older adults’ compliance with fall prevention measures [48]. The current study revealed that a robust social support network significantly enhances older adults’ self-management abilities in preventing falls. Notably, family support serves as a primary motivation for older adults to engage in fall prevention within their communities. A nationwide survey conducted in China [49] revealed a high demand for community services among older adults, particularly in the areas of healthcare, daily care, and spiritual care. However, the supply of these services remains inadequate, resulting in a supply-demand imbalance. These findings align with the results of the present study, which highlights limited community engagement with older adults and the absence of a comprehensive support network for fall prevention. This may be attributed to the large population of residents in the community and the cumbersome procedures involved in handling community affairs. Consequently, it is imperative for the responsible governmental entity to reduce the associated burden and increase support, enabling the community to play a pivotal role in promoting self-managed fall prevention among older adults [50]. The prevention of falls among older adults is a societal responsibility that requires the participation of all stakeholders. To this end, the responsible governmental entity must establish an age-friendly environment, enhance the construction of suitable living environments, and optimize supportive environments specifically tailored to meet the needs of older adults [51].

It is noteworthy that the prevalence of falls among Chinese older adults residing in rural areas (22%) was comparable to that observed in urban areas (18%) [52]. Chinese rural older adults have traditionally engaged in agricultural labour for prolonged periods, often resulting in a limited awareness regarding fall prevention [53]. Agricultural production activities can potentially hasten the degradation of musculoskeletal system functions, thus increasing the risk of falls [52]. Among Chinese rural older adults, physical activity levels and moderate-to-vigorous intensity exercise have been associated with an increased risk of falls [54]. Consequently, the implementation of fall prevention strategies among rural older adults in China is equally crucial. Our study primarily focuses on community-dwelling older adults, who are relatively less impacted by physiological changes, aiming to serve as a guiding light for self-managed fall prevention efforts. Subsequently, we aim to extend the scope of our research to encompass rural older adults and investigate the practical measures they can adopt to enhance self-managed fall prevention, ultimately aiming to reduce the overall occurrence of falls.

## Limitations

This study acknowledges its several limitations. Firstly, due to constraints in human and material resources, the investigation and evaluation were confined to older adults residing in urban communities in Fuzhou. Consequently, the findings may not be generalizable to other settings. Future research should aim to broaden the scope of the investigation to include rural and urban areas across multiple provinces to facilitate comparisons and explore the perspectives of older adults in different regions on self-managed fall prevention, ultimately leading to the development of more effective measures for this population. Secondly, the study did not differentiate between older adults based on their disease types. Future studies would benefit from interviewing older adults with various high-risk fall diseases to gain a deeper understanding of the influencing factors and coping strategies related to self-managed fall prevention. This knowledge would significantly contribute to the design of more tailored and effective interventions for this vulnerable group. Finally, while conducting interviews about self-managed fall prevention among Chinese older adults, we primarily reviewed strategies relevant to China. The promotion and application of the research results remain to be verified and validated.

## Conclusions

This study is a qualitative investigation examining the perspectives of community-dwelling older adults regarding self-managed fall prevention in their daily lives. The findings reveal that these older adults possess a sense of self-managed fall prevention, believing that their own strength plays a crucial role in preventing falls. However, it is noteworthy that they lack knowledge in this domain, have limited access to information, and cannot independently guarantee the authenticity of professional and authoritative sources. Additionally, they experience limited social support. These insights provide valuable references for individuals, families, and community workers to prioritize self-managed fall prevention and its associated countermeasures for older adults. Future research can further explore programs tailored for self-managed fall prevention among older adults, aiming to develop coping strategies that enhance their current situation.

## Supplementary Material

Supplemental Material

## Data Availability

The datasets generated and/or analyzed during the current study are not publicly available due this article is part of the author’s master’s thesis with a confidentiality period of 2 years, and other related papers have not yet been published. The datasets were not suitable for publication now but are available from the corresponding author upon reasonable request.
