# Supplementary material for: Self-management experiences in fall prevention among community-dwelling older adults in China: a descriptive qualitative study
Source: Ann Med. 2024 Sep 25;56(1):2392878. doi: 10.1080/07853890.2024.2392878 (PMC11425688; doi:10.1080/07853890.2024.2392878)
Supplement: Supplemental Material [file IANN_A_2392878_SM0603.zip › Supp_Data/Implications for Rehabilitation.docx]

**Self-management experiences in fall prevention among community-dwelling older adults in China: A descriptive qualitative study**

- With the increase of age, the incidence of falls gradually increases, which can bring a heavy economic and care burden to families and society.
- Fall prevention among community-dwelling older adults is one of the primary measures to achieve healthy aging, in which older adults can play an important role.
- The effect of self-management is better than passive participation in disease management.
- Self-management of fall prevention takes older adults themselves as the main body, emphasizes their responsibility in maintaining their health and their confidence in active response, and pays attention to the skills training and behavior change of older adults, which is a new idea for older adults to prevent falls.
