# Supplementary material for: Self-management experiences in fall prevention among community-dwelling older adults in China: a descriptive qualitative study
Source: Ann Med. 2024 Sep 25;56(1):2392878. doi: 10.1080/07853890.2024.2392878 (PMC11425688; doi:10.1080/07853890.2024.2392878)
Supplement: Supplemental Material [file IANN_A_2392878_SM0603.zip › Supp_Data/Supplementary File 2 Interview Guide.docx]

**Supplementary File 2**

**Interview Guide**

(a) What do you think about falling?

(b) What impact if you fall?

(c) What do you think about self-management for fall prevention?

(d) What promoting factors or obstructive factors affect your self-management for fall prevention?

(e) What actions to engage in self-managed fall prevention?

(f) How do you feel about taking steps to self-management for fall prevention?

(g) What else would you like others or outsiders to do to help you to self-management for fall prevention?
